# SNAI2 upregulation is associated with an aggressive phenotype in fulvestrant-resistant breast cancer cells and is an indicator of poor response to endocrine therapy in estrogen receptor-positive metastatic breast cancer

**DOI:** 10.1186/s13058-018-0988-9

**Published:** 2018-06-19

**Authors:** Carla L. Alves, Daniel Elias, Maria B. Lyng, Martin Bak, Henrik J. Ditzel

**Affiliations:** 1 Department of Cancer and Inflammation Research, Institute of Molecular Medicine, University of Southern Denmark, J.B. Winsløwsvej 25, 5000 Odense C, Denmark; 2 Department of Pathology, Odense University Hospital, 5000 Odense, Denmark; 3 Department of Oncology, Odense University Hospital, 5000 Odense, Denmark; 4 Academy of Geriatric Cancer Research (AgeCare), Odense University Hospital, 5000 Odense, Denmark

**Keywords:** Endocrine resistance, Epithelial-mesenchymal transition, Estrogen receptor-positive breast cancer, Fulvestrant, SNAI2

## Abstract

**Background:**

Endocrine resistance in estrogen receptor-positive (ER+) breast cancer is a major clinical problem and is associated with accelerated cancer cell growth, increased motility and acquisition of mesenchymal characteristics. However, the specific molecules and pathways involved in these altered features remain to be detailed, and may be promising therapeutic targets to overcome endocrine resistance.

**Methods:**

In the present study, we evaluated altered expression of epithelial-mesenchymal transition (EMT) regulators in ER+ breast cancer cell models of tamoxifen or fulvestrant resistance, by gene expression profiling. We investigated the specific role of increased SNAI2 expression in fulvestrant-resistant cells by gene knockdown and treatment with a SNAIL-p53 binding inhibitor, and evaluated the effect on cell growth, migration and expression of EMT markers. Furthermore, we evaluated SNAI2 expression by immunohistochemical analysis in metastatic samples from two cohorts of patients with breast cancer treated with endocrine therapy in the advanced setting.

**Results:**

SNAI2 was found to be significantly upregulated in all endocrine-resistant cells compared to parental cell lines, while no changes were observed in the expression of other EMT-associated transcription factors. SNAI2 knockdown with specific small interfering RNA (siRNA) converted the mesenchymal-like fulvestrant-resistant cells into an epithelial-like phenotype and reduced cell motility. Furthermore, inhibition of SNAI2 with specific siRNA or a SNAIL-p53 binding inhibitor reduced growth of cells resistant to fulvestrant treatment. Clinical evaluation of SNAI2 expression in two independent cohorts of patients with ER+ metastatic breast cancer treated with endocrine therapy in the advanced setting (*N* = 86 and *N* = 67) showed that high SNAI2 expression in the metastasis correlated significantly with shorter progression-free survival on endocrine treatment (*p* = 0.0003 and *p* = 0.004).

**Conclusions:**

Our results suggest that SNAI2 is a key regulator of the aggressive phenotype observed in endocrine-resistant breast cancer cells, an independent prognostic biomarker in ER+ advanced breast cancer treated with endocrine therapy, and may be a promising therapeutic target in combination with endocrine therapies in ER+ metastatic breast cancer exhibiting high SNAI2 levels.

**Electronic supplementary material:**

The online version of this article (10.1186/s13058-018-0988-9) contains supplementary material, which is available to authorized users.

## Background

Approximately 80% of all breast tumors are positive for estrogen receptor (ER+), which is an indicator of potential responsiveness to endocrine therapy both in the adjuvant and advanced settings [1]. Despite the efficacy of endocrine therapy for treatment of ER+ breast cancer, a significant number of patients develop resistance to these drugs. There is considerable evidence suggesting that acquisition of endocrine resistance is accompanied by accelerated tumor growth and increased metastatic propensity, and is associated with morphological changes characteristic of cells undergoing epithelial-mesenchymal transition (EMT) [2]. However, key questions remain regarding the central molecules controlling the EMT process during development of endocrine resistance, which may be promising therapeutic targets in combination with endocrine therapy.

EMT is a complex process characterized by loss of epithelial features, such as downregulation of the E-cadherin and occludins, and acquisition of mesenchymal properties, including upregulation of vimentin and fibronectin, and cytoskeleton reorganization [3]. EMT has been associated with increased cell migration capacity and invasiveness, and is a prominent hallmark of cancer progression [4, 5]. Epithelial tumor cells may acquire a mesenchymal-like phenotype to facilitate migration and invasion and then possibly reverse to an epithelial state through mesenchymal-epithelial transition (MET) to form organized tumorigenic nodules at the lodgment sites [6]. EMT and MET are regulated by signals from the stroma associated with tumors, such as transforming growth factor (TGF)-β, and by a series of EMT-inducing transcription factors, including SNAI1, SNAI2, TWIST, ZEB1 and ZEB2 [7].

The role of EMT in endocrine resistance was first reported in studies on ER-depleted breast cancer cells, which were found to convert the non-invasive epithelial features into a mesenchymal-like phenotype with invasive characteristics [6, 8]. Moreover, EMT has been shown to mediate endocrine resistance through the action of EMT transcription factors. SNAI family members were found to directly repress ER [9, 10] and enhance the anti-apoptotic behavior of cancer cells, contributing to resistance to therapy [11]. A large body of evidence supports the importance of EMT in sustaining cancer stem cells (CSCs), which can be intrinsically resistant to treatment [12]. Furthermore, several growth factor receptors, such as epidermal growth factor receptor (EGFR), insulin-like growth factor 1 receptor (IGF-1R) and fibroblast growth factor 1 receptor (FGFR1), which are involved in the EMT process, are highly expressed in ER- breast tumor cells, supporting the link between EMT and insensitivity to endocrine therapy [2]. The emerging role of EMT as a mediator of endocrine resistance in breast cancer has raised interest in therapeutic strategies based on reversing EMT to prevent tumor progression and re-sensitizing tumor cells to endocrine therapy [13]. One promising pharmacological approach involves the development of specific inhibitors of EMT-associated transcription factors to therapeutically inhibit EMT induction or target the mesenchymal cell type [14].

In this study, we investigated the altered expression of various EMT regulators in MCF-7-based breast cancer cell models of endocrine resistance by gene array. We observed upregulation of SNAI2 in fulvestrant-resistant and tamoxifen-resistant cells compared to the parental cell lines, while other EMT-associated transcription factors were not altered. Inhibition of SNAI2 induced epithelial characteristics, reduced cell motility, and impaired growth of fulvestrant-resistant breast cancer cells. High levels of SNAI2 in ER+ metastatic tumor samples from two cohorts of patients treated with endocrine therapy in the advanced setting correlated significantly with poor clinical outcome. Our findings indicated SNAI2 as an independent prognosis biomarker in ER+ metastatic breast cancer patients treated with endocrine therapy and a potential novel therapeutic target that may contribute to reversing EMT and re-sensitizing breast cancer cells to endocrine therapy.

## Methods

### Cell lines and culture conditions

The original MCF-7 cell line was obtained from the Breast Cancer Task Force Cell Culture Bank, Mason Research Institute. MCF-7 cells were gradually adapted to grow in low serum concentration [15], and this subline, MCF-7/S0.5, was used to establish two fulvestrant-resistant cell models, MCF-7/182R (including 182R-1 and 182R-6 cell lines) and MCF-7/164R (including 164R-1 and 164R-4 cell lines), by extended treatment with 100 nM of fulvestrant (ICI 182,780) or ICI 164,384, respectively [16]. Tamoxifen-resistant (TamR) cell lines, including TamR-1, TamR-4, TamR-7 and TamR-8 cells, were established from MCF-7/S0.5 by long-term treatment with 1 μM of tamoxifen [17]. The MCF-7/S0.5 cell line was routinely propagated in phenol red-free Dulbecco’s modified Eagle medium (DMEM)/F12 (Gibco) supplemented with 1% glutamine (Gibco), 1% heat-inactivated fetal bovine serum (FBS; Gibco) and 6 ng/ml insulin (Sigma-Aldrich). Fulvestrant-resistant and tamoxifen-resistant cell lines were maintained in the same growth medium as MCF-7/S0.5 supplemented with 100 nM fulvestrant (Tocris) or 1 μM tamoxifen (Sigma-Aldrich), respectively. Cells were grown in a humidified atmosphere of 5% CO_2_ at 37 °C and the growth medium was renewed every second or third day. To reduce variability between experiments, cells were maintained at low passage numbers (< 10 passages) throughout the experiments. All cell lines underwent DNA authentication using Cell ID™ System (Promega) before the described experiments to ensure consistent cell identity.

### Global gene expression profiling

MCF-7/S0.5, tamoxifen-resistant and fulvestrant-resistant cell lines were grown to 70–80% confluence for total RNA purification using a RNA kit (Qiagen) and arrayed separately in Affymetrix Gene Chip® Human Genome U133 plus 2.0 arrays (Affymetrix), as described [18]. Data were analyzed using Partek Genomic Suite (Partek Inc.). Genes from the data set that exhibited twofold or greater alteration in expression and false discovery rate (FDR) cutoff < 0.05 were considered as significantly altered regulated.

### RNA isolation and reverse transcription (RT)-quantitative (q)PCR (RT-qPCR)

Total RNA was extracted using Isol-Lysis Reagent, TRIzol® (Life technologies) followed by chloroform and isopropyl alcohol (Sigma-Aldrich) for separation and precipitation of RNA. Concentration and purity were measured using the NanoDrop-1000 spectrophotometer (Saveen). Complementary DNA (cDNA) was synthesized using RevertAid Premium Reverse Transcriptase kit (Fermentas). Relative quantification of gene expression was performed using SYBR® Green PCR Mastermix (Applied Biosystems) according to manufacturer’s instructions. The following primers purchased from Qiagen were used: *SNAI2* (QT00044128), *CDH1* (QT00080143), and *PUM1* (QT00029421) QuantiTect® Primer. *PUM1* was used as the reference gene for normalization. RT-qPCR reactions were performed on a StepOnePlus™ Real-Time PCR system from Applied Biosystems and data were obtained from StepOne Software Version 2.1. Relative expression levels were calculated using the comparative threshold method [19].

### Western blotting

Whole cell extracts were obtained using radioimmunoprecipitation assay (RIPA) buffer (50 mM Tris HCl (pH 8), 150 mM NaCl (pH 8), 1% IgePAL 630, 0.5% sodium dioxycholate, 0.1% SDS) containing protease and phosphatase inhibitors (Roche). The protein concentration of the lysate samples was determined using Pierce bicinchoninic acid (BCA) Protein Assay Kit (Thermo Fisher Scientific) and the optical density (OD) was measured at 562 nm in the microplate reader Sunrise^TM^ 500 ELISA-reader (Tecan). 10–20 μg of total protein lysate was loaded on a 4–20% SDS-PAGE gel (Biorad) under reducing conditions and electroblotted onto a polyvinylidene difluoride (PVDF) transfer membrane. Prior to primary antibody incubation, membranes were blocked in Tris-buffered saline (TBS), 0.1% Tween-20 (Sigma-Aldrich) containing 5% non-fat dry milk powder (Sigma-Aldrich) or 5% bovine serum albumin (Sigma-Aldrich). The following antibodies were used according to the manufacturer’s protocol: anti-E-cadherin (#3195, Cell Signaling), anti-SNAI2 (#9585, Cell Signaling); anti-vimentin (#6630, Sigma-Aldrich); anti-ERα antibody (#9101, Thermo Fisher Scientific); anti-SOX2 (#AF2018, R&D Systems); anti-β-actin (#6276, Abcam) as loading control; horseradish peroxidase (HRP)-conjugated goat anti-mouse (#P0447, Dako); HRP-conjugated goat anti-rabbit (#P0448, Dako); HRP-conjugated donkey anti-goat (#sc-2020, Santa Cruz Biotechnology). The membrane was developed with Enhanced Chemiluminescence (ECL) Prime Western Blotting Detection Reagents (GE Healthcare) and visualized using the Fusion-Fx7–7026 WL/26MX instrument (Vilbaer).

### siRNA-mediated gene knockdown

Cells were transfected with siRNA against SNAI2 (s13127; Life Technologies) or SOX2 (D-011778-01; Dharmacon) using an Electroporation Ingenio kit (Mirus Bio) in a Nucleofector™ II device (Amaxa, Lonza) or Lipofectamine 3000 reagent (Thermo Fischer Scientific), respectively, according to manufacturers’ instructions. Mission siRNA Universal Negative Control (SIC001) (Sigma-Aldrich) was used as control. Transfected cells were seeded in 24-well plates (5 × 10^4^ cells/well) to evaluate gene knockdown efficiency 48 h following transfection, by RT-qPCR. Transfected cells were seeded in T25 flasks (5 × 10^5^ cells) and incubated for 96 h to assess protein expression by western blotting.

### Cell growth assay

Transfected cells were seeded (2.5–5 × 10^4^ cells/well) in 24-well plates and incubated for 24 and 96 h at 37 °C in 5% CO_2_ for evaluation of cell growth using crystal violet-based colorimetric assay [20]. For growth assays with the chemical inhibitor, cells were seeded (3 × 10^4^ cells/well) in 24-well plates in the presence of 3 μM SNAIL-p53 binding inhibitor GN25 (Millipore) or its solvent (DMSO, Sigma-Aldrich), and cell growth was measured 72 h after seeding using crystal violet-based colorimetric assay. The OD was analyzed at 570 nm in a Sunrise™ 500 absorbance reader (Tecan).

### Cell migration assay

A total of 1 × 10^5^ cells, starved overnight, were harvested in serum-free medium and seeded in the upper chamber of 8-μm-pore polystyrene membrane chamber-insert Transwell® apparatus (Corning, Costar) in 24-well plates with 10% FBS medium, according to the manufacturer’s instructions. Cells were incubated for 96 h at 37 °C in 5% CO_2_. Cells on the top surface of the insert were removed with a cotton swab, and cells that migrated to the bottom face of the insert were fixed and stained with crystal violet in methanol solution. To determine the number of migrated cells, five random fields were used to count cells at the microscope. To determine the total number of cells that migrated in one insert, the average number of cells counted was divided by the area of the microscope viewing field and then multiplied by the entire area of the Transwell insert (0.3 cm^2^). Normalization of migration according to growth rate was performed using crystal violet staining.

### Cell invasion assay

Cell invasion was evaluated using a QCM ECMatrix 24-well kit (Chemicon ECM550) according to the manufacturer’s instructions. Cells were seeded in serum-free medium in the upper chamber of an insert in 24-well plates with 10% FBS medium, and incubated for 96 h at 37 °C in 5% CO_2_. Invading cells were detached, lysed, stained with dye, and measured by fluorescent light emission (480 nm/520 nm) using a Victor3™ 1420 counter (Perkin Elmer Wallac). Fluorescent measurements were reported as relative fluorescent unit (RFU) values. Light emission was normalized to cell growth rate measured by crystal violet colorimetric assay.

### Immunocytochemical analysis

MCF-7/S0.5 and fulvestrant-resistant cells were fixed in 4% formalin, paraffin-embedded, and mounted in 4-μm sections on glass slides. Antigen retrieval was performed by boiling sections in T-EG solution/TRS buffer (Dako). Sections were incubated with anti-SOX2 antibody (#AF2018, R&D Systems) for one hour at room temperature. PowerVision Poly-HRP was used as the detection system. Microscopy of cells was performed using a Leica DMLB microscope (× 100 or × 200/numerical aperture (NA) 1.25, Leica Microsystems) using LasV3.6 acquisition software.

### Clinical samples

Formalin-fixed, paraffin-embedded (FFPE) metastatic tumor samples from patients with ER+ breast cancer treated with endocrine therapy in the advanced setting were selected by database extraction from the archives of the Department of Pathology at Odense University Hospital (OUH) encompassing the period 2004–2013 (*N* = 165; cohort 1) and 2013–2016 (*N* = 128; cohort 2). Patients eligible for inclusion were those with ER+ breast cancer with metastatic disease, who had undergone surgery or biopsy at OUH, and for whom complete clinical information and pathological verification that the metastatic lesion was of breast cancer origin were available. Exclusion criteria were competing cancer(s), cytological biopsies, or insufficient material in the FFPE block. These parameters yielded 86 (cohort 1) and 67 (cohort 2) metastatic lesions from patients with advanced breast cancer treated with endocrine therapy. The metastatic biopsies used for evaluation of SNAI2 expression were obtained prior to treatment with endocrine therapy. Tumors were defined as ER+ if ≥ 1% of the tumor cells stained positive.

### Immunohistochemical staining

Whole FFPE sections of metastatic lesions were incubated with anti-SNAI2 (#sc-15391, Santa Cruz Biotechnology) and immunostained using the HRP-conjugated PowerVision+™ system on the autostainer TechMateTM 500 (Dako), as described [21]. A Leica DMLB microscope (× 100/numerical aperture 1.25, Leica Microsystems) and LasV3.6 acquisition software were used for tissue microscopy. Evaluation of the staining was performed by an experienced breast pathologist in a blinded setup. SNAI2 expression was observed in the cell nucleus and cytoplasm and tumors were scored based on the staining intensity score (0–3). The cutoff value for high versus low SNAI2 (intensity score = 2) was determined and optimized in cohort 1 employing the web-based tool Cutoff Finder [22] with the “survival significance” function, and the same cutoff was then applied to cohort 2.

### Clinical endpoints

Progression-free survival (PFS) was defined as the time from the date of initiation of endocrine treatment until disease progression within 5 years. Patients without progression within a 5-year period were censored at the date of database retrieval from the registry or 5 years from the date of endocrine treatment initiation, whichever came first.

### Statistical analysis

One-way analysis of variance (ANOVA) was used based on twofold or greater change in expression and a FDR of < 0.05 to select genes differentially expressed between MCF-7-based endocrine-resistant and endocrine-sensitive cell lines. The two-tailed *t* test was used to compare data between groups from RT-qPCR, cell growth and migration assays. Association between SNAI2 expression and patient clinicopathological parameters was determined by Fisher’s exact and the chi-square (*χ*^2^) test. Multivariate analysis was performed using a Cox proportional hazard regression model to assess the adjusted hazard ratio (HR) of PFS by SNAI2 expression and clinicopathological characteristics, including age at metastasis, site of relapse and human epidermal growth factor receptor 2 (HER2) status. Survival curves were generated using Kaplan-Meier estimates from the log-rank test to evaluate the correlation between SNAI2 expression and PFS. STATA v14.0 (STATACorp) and GraphPad Prism v5.0 (GraphPad Software, Inc.) were used for statistical analysis*. P* values < 0.05 were considered statistically significant.

## Results

### The EMT transcription regulator SNAI2 is upregulated in endocrine-resistant breast cancer cells

**Fig. 1:**
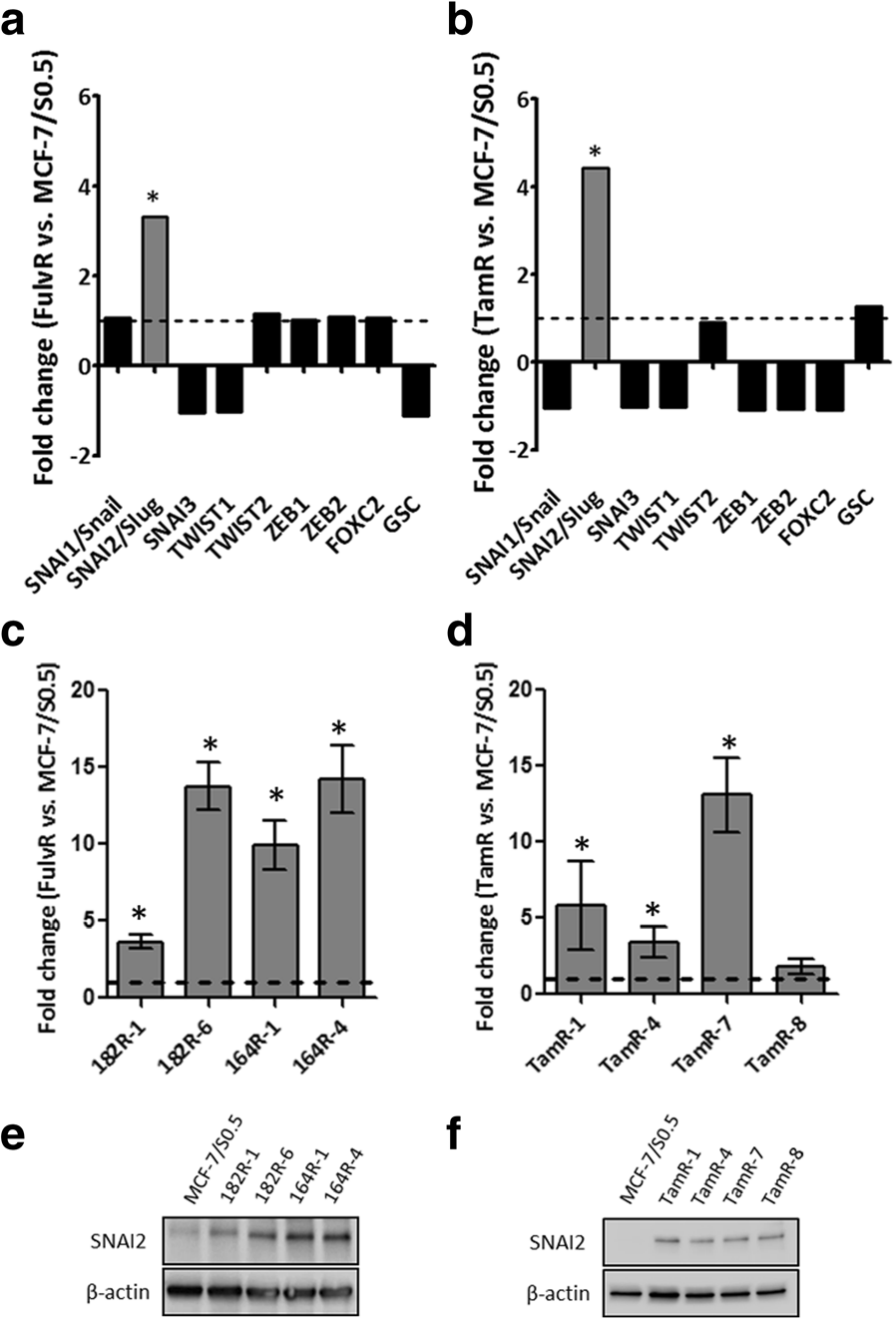
Upregulation of SNAI2 in endocrine-resistant cells compared to parental endocrine-sensitive cell lines. Evaluation of the gene expression of epithelial-mesenchymal transition (EMT) transcription regulators in MCF-7-based fulvestrant-resistant (FulvR) vs. fulvestrant-sensitive (**a**) and tamoxifen-resistant (TamR) vs. tamoxifen-sensitive (**b**) cells by gene array. Verification of altered expression of SNAI2 in four fulvestrant-resistant (**c**) and four tamoxifen-resistant cell lines (**d**), by reverse transcription (RT)-quantitative (q)PCR (RT-qPCR). Gene expression was normalized using *PUM1* and depicted as the relative expression in endocrine-resistant vs. endocrine-sensitive cells: *p < 0.05. Data are shown with error bars representing mean ± standard deviation. SNAI2 protein expression alteration in the same fulvestrant-resistant (**e**) and tamoxifen-resistant cell lines (**f**), as determined by western blotting. β-actin was used as loading control. A representative of three independent experiments is shown

To identify EMT-associated genes involved in the endocrine-resistant phenotype of breast cancer cells, we evaluated whether several EMT regulators exhibited altered expression in our gene expression profiling data from MCF-7-based fulvestrant-resistant and tamoxifen-resistant cell lines [18, 23]. Among the transcription factors involved in the regulation of EMT, SNAI2 was found to be significantly upregulated in fulvestrant-resistant (FulvR) and tamoxifen-resistant (TamR) cells compared to their parental cell lines (twofold or greater alteration in expression, *p* < 0.05) (Fig. 1a and b). Altered expression of SNAI2 was verified in two fulvestrant-resistant cell line models (182R and 164R), each containing two cell lines, and four tamoxifen-resistant cell lines, at messenger RNA (mRNA) (Fig. 1c and d) and protein levels (Fig. 1e and f) using RT-qPCR and western blotting, respectively. We previously demonstrated the functional importance of SNAI2 in tamoxifen resistance [24], therefore the present study focused on the role of this EMT-associated transcription factor in the mechanisms of resistance to fulvestrant.

### Fulvestrant-resistant cells show increased motility and higher expression of mesenchymal markers compared to fulvestrant-sensitive cell lines

**Fig. 2:**
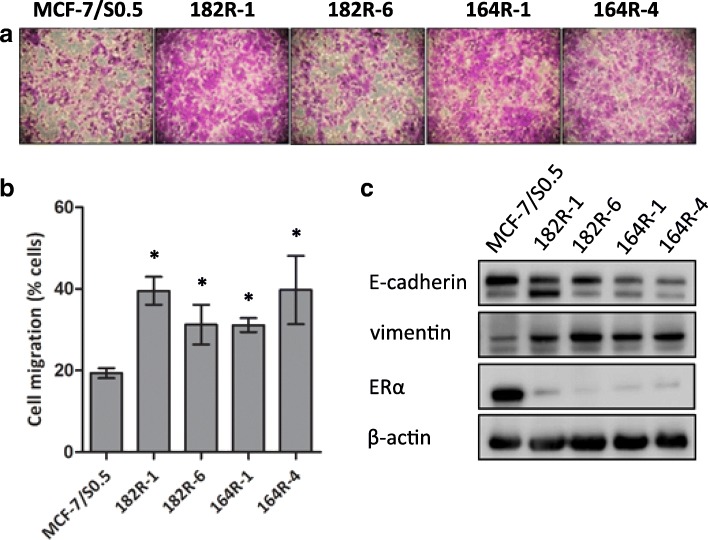
Fulvestrant-resistant cells exhibit a more motile mesenchymal phenotype compared to parental fulvestrant-sensitive cells. The motility of MCF-7-based fulvestrant-sensitive and fulvestrant-resistant cells was evaluated by a transwell assay. **a** Representative micrographs (purple-stained cells, × 20 magnification) and **b** column diagram analysis of the percentage of cells that migrated through the membrane: *p < 0.05. Data are shown with error bars representing mean ± standard deviation. **c** Protein expression levels of E-cadherin, vimentin and estrogen receptor (ER)α by western blotting. β-actin was used as loading control. A representative of three independent experiments is shown

To investigate whether SNAI2 upregulation in fulvestrant-resistant cells is associated with a mesenchymal phenotype, we evaluated cell migration, invasion, and expression of EMT markers in the two fulvestrant-resistant models. All four fulvestrant-resistant cell lines had significantly greater ability to migrate compared with fulvestrant-sensitive cell lines (Fig. 2a and b). However, neither fulvestrant-resistant nor fulvestrant-sensitive cells were able to invade extracellular matrix (ECM)-coated membrane (data not shown). Further, fulvestrant-resistant cells exhibited higher expression of vimentin and lower expression of E-cadherin, consistent with a mesenchymal phenotype, whereas parental fulvestrant-sensitive cells exhibited epithelial-like features, including high E-cadherin and low vimentin expression (Fig. 2c). The expression of ER was markedly reduced, but still present, in all fulvestrant-resistant cells compared to the parental cell line (Fig. 2c).

### SNAI2 knockdown restores epithelial-like features and impairs growth of fulvestrant-resistant breast cancer cells

**Fig. 3:**
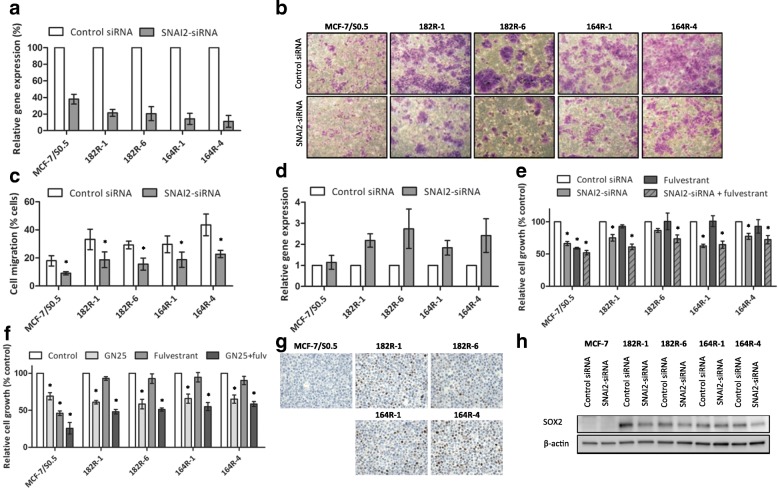
Inhibition of SNAI2 induces epithelial characteristics and impairs growth of fulvestrant-resistant breast cancer cell lines. **a** MCF-7-based fulvestrant-resistant and parental sensitive cells were transfected with small interfering RNA (siRNA) against SNAI2 leading to a reduction at the messenger RNA (mRNA) level, as evaluated by reverse transcription (RT)-quantitative (q)PCR (RT-qPCR). SNAI2 knockdown resulted in a significant reduction in the migration ability of both fulvestrant-resistant and fuvestrant-sensitive cells, as depicted in **b** representative micrographs (purple-stained cells, × 20 magnification) and **c** by column diagram analysis of the percentage of cells that migrated following SNAI2 knockdown. **d** E-cadherin mRNA expression after SNAI2 silencing determined by RT-qPCR. Gene expression was normalized using *PUM1*. **e** Effect of SNAI2 knockdown on growth of fulvestrant-resistant and parental sensitive cells, as measured by crystal violet-based colorimetric assay. **f** Cell growth of fulvestrant-resistant and parental sensitive cells following treatment with the selective p53-SNAIL binding inhibitor (GN25), fulvestrant, the two drugs in combination or vehicle (control), as measured by crystal violet-based colorimetric assay: *p < 0.05. Data are shown with error bars representing mean ± standard deviation. **g** Immunocytochemical analysis of SOX2 protein in formalin-fixed paraffin-embedded fulvestrant-resistant and fulvestrant-sensitive cells (× 20 magnification). **h** Protein expression levels of SOX2 following SNAI2 knockdown determined by western blotting. β-actin was used as loading control. A representative of three independent experiments is shown

To evaluate the role of SNAI2 in the control of EMT characteristics and resistance to fulvestrant, we performed gene knockdown studies with specific siRNA targeting SNAI2 in the two fulvestrant-resistant breast cancer cell models. Transient transfection of siRNA against SNAI2 led to efficient downregulation of SNAI2 in both fulvestrant-resistant cells and the parental MCF-7 cell line, as determined by RT-qPCR (Fig. 3a). Protein levels of SNAI2 following SNAI2 knockdown were determined by western blotting and are shown in Additional file 1: Figure S1A. Migration of fulvestrant-resistant cells following SNAI2 downregulation was significantly reduced compared with cells transfected with control siRNA (Fig. 3b and c). Furthermore, E-cadherin mRNA expression was increased in fulvestrant-resistant cells following SNAI2 knockdown, as determined by RT-qPCR (Fig. 3d), supporting that there is alteration to a more epithelial-like phenotype. Protein levels of E-cadherin following SNAI2 knockdown were determined by western blotting and are shown in Additional file 1: Figure S1B. Additionally, inhibition of SNAI2 by siRNA-mediated knockdown or treatment with SNAIL-p53 binding inhibitor (GN25) reduced growth of fulvestrant-resistant cells (Fig. 3e and f, respectively). The effect of SNAI2 inhibition in reducing fulvestrant-sensitive cell growth was comparable to that of fulvestrant treatment alone (Fig. 3e and f).

As SNAI2 has been implicated in breast CSCs by controlling SOX2 transcription [25], we evaluated SOX2 levels in FFPE fulvestrant-resistant and parental sensitive cell lines and found that SOX2 was upregulated in fulvestrant-resistant compared to fulvestrant-sensitive cells (Fig. 3g). Next, we evaluated the effect of SNAI2 knockdown in SOX2 levels and observed a marked reduction in SOX2 protein expression in three of the four fulvestrant-resistant cell lines (Fig. 3h), suggesting that upregulation of SOX2 in fulvestrant-resistant cells may be mediated by SNAI2. Finally, we evaluated the relevance of SOX2 in fulvestrant resistance by siRNA-mediated knockdown (Additional file 2: Figure S2). We found that reduction of SOX2 expression resulted in decreased fulvestrant-resistant cell growth comparable to the effect of SNAI2 inhibition, which suggests that the role of SNAI2 in controlling growth of resistant cells might be dependent on regulation of SOX2 expression.

### SNAI2 expression strongly correlates with clinical outcome in patients with ER+ advanced breast cancer

**Fig. 4:**
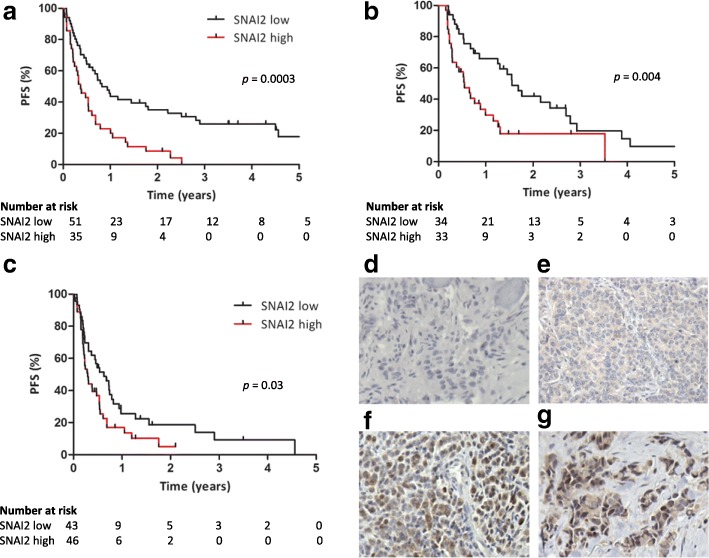
SNAI2 expression correlates with progression-free survival (PFS) in patients with estrogen receptor (ER)+ metastatic breast cancer treated with endocrine therapy. Kaplan-Meier plots evaluating PFS according to expression of SNAI2 in ER+ metastatic lesions from **a** an initial and **b** a second cohort of patients with breast cancer treated with endocrine therapy in the advanced setting. **c** Survival analysis of PFS according to SNAI2 levels in fulvestrant-treated patients from cohorts 1 and 2. A two-sided *p* value (*p < 0.05) was calculated using log-rank testing. Representative micrographs of breast cancer metastasis sections showing low SNAI2 expression (**d** and **e**) or high SNAI2 expression (**f** and **g**) (× 20 magnification)

To investigate the clinical relevance of SNAI2, we evaluated the expression of this protein by immunohistochemical analysis in full sections of ER+ metastatic lesions from an initial cohort of postmenopausal patients treated with endocrine treatment in the advanced setting (*N* = 86), including patients treated with fulvestrant, tamoxifen, and aromatase inhibitors. Clinical and pathological characteristics of this cohort are shown in Table 1. Survival analysis showed that patients with tumors expressing high levels of SNAI2 exhibited significantly shorter PFS on endocrine therapy (median time to progression 4.41 months vs. 9.90 months, *p* = 0.0003) (Fig. 4a). To validate the results from the initial cohort, we analyzed a second cohort of postmenopausal patients with ER+ metastatic breast cancer treated with endocrine treatment in the advanced setting (*N* = 67), including patients treated with fulvestrant, tamoxifen, and aromatase inhibitors. Clinical and pathological characteristics of the second cohort are also shown in Table 1. Analysis of the data from this cohort confirmed significant correlation between high SNAI2 expression and shorter PFS in patients on endocrine therapy (6.57 months vs. 18.67 months, *p* = 0.004) (Fig. 4b). Furthermore, we tested the correlation between SNAI2 expression in metastasis and PFS in patients treated with fulvestrant, from cohort 1 (*N* = 45) and cohort 2 (*N* = 44) (Additional file 3: Figure S3A and B, respectively). Although there were no statistically significant results, likely due to the small number of patients in individual analysis of the two cohorts, there was separation of the Kaplan-Meier curves and the median time to progression was shorter in patients with SNAI2-high than with SNAI2-low metastasis in both cohorts (cohort 1, median time to progression 4.16 vs. 7.56 months, respectively, *p* = 0.07; cohort 2, median time to progression of 3.35 vs. 8.12 months, respectively, *p* = 0.14). To increase the sample size, we performed Kaplan-Meier estimates and the log-rank test in fulvestrant-treated patients from both cohorts combined (*N* = 89), which showed shorter PFS in patients with metastasis exhibiting high SNAI2 compared with those with metastasis expressing low SNAI2 (median time to progression of 3.52 vs. 7.56 months, respectively, *p* = 0.03) (Fig. 4c). Representative micrographs of breast cancer sections showing low SNAI2 expression (staining intensity score 0 and 1) or high SNAI2 expression (staining intensity score 2 and 3) are presented in Fig. 4d–g. None of the available clinicopathological characteristics of either primary or metastatic tumors correlated significantly with SNAI2 levels (Table 1). Cox proportional hazard regression analysis of PFS according to SNAI2 level and clinicopathological characteristics of the metastatic disease, including age, site of relapse and HER2 status (Table 2), showed that SNAI2 was independently prognostic of PFS on endocrine therapy in both cohorts (cohort 1, HR 2.11, 95% CI of the ratio, 1.21–3.66, *p* = 0.008; cohort 2, HR 1.92, 95% CI of the ratio, 1.03–3.59, *p* = 0.04).

**Table 1:** Clinical and pathological characteristics of patients with estrogen receptor (ER)+ advanced breast cancer according to SNAI2 level

	Cohort 1				Cohort 2			
Parameters	SNAI2 low	SNAI2 high	Number	*p* ^a^	SNAI2 low	SNAI2 high	Number	*p* ^a^
Age at primary tumor								
≤ 50 years	8	6	14	0.86	14	7	21	0.11
> 50 years	43	29	72		20	26	46	
Age at metastatic disease								
≤ 50 years	3	1	4	0.64	7	1	8	0.05
> 50 years	48	34	82		27	32	59	
Size (mm) of primary tumor								
≤ 20	18	12	30	0.24	12	10	22	0.34
> 20 to ≤ 50	18	13	31		11	17	28	
> 50	1	4	5		2	2	4	
Unknown	14	6	20		9	4	13	
Nodal status of primary tumor Number of positive lymph nodes								
0	11	10	21	0.46	7	6	13	0.37
1–3	16	10	26		8	10	18	
> 3	12	11	23		7	11	18	
Unknown	12	4	16		12	6	18	
Grade of primary tumor								
I	8	9	17	0.06	6	6	12	0.06
II	12	15	27		7	16	23	
III	9	2	11		5	1	6	
Unknown	22	9	31		16	10	26	
HER2 status of metastasis								
Normal	41	30	71	0.62	29	31	60	0.11
Amplified	4	1	5		1	2	3	
Unknown	6	4	10		4	0	4	
Dominant site of relapse								
Soft tissue	38	23	61	0.65	15	17	32	0.17
Bone	8	8	16		15	8	23	
Viscera	5	4	9		4	8	12	
Total number	51	35	86		34	33	67	

*HER2* human epidermal growth factor receptor 2

^a^χ2 or Fisher’s exact test

**Table 2:** Regression analysis of progression-free survival according to SNAI2 level and clinicopathological characteristics

	Cohort 1		Cohort 2	
Variable	Hazard ratio (95% CI)	*p*	Hazard ratio (95% CI)	*p*
SNAI2 level	2.11 (1.21–3.66)	0.008	1.92 (1.03–3.59)	0.04
Age at metastasis	2.19 (0.37–12.95)	0.39	0.90 (0.33–2.49)	0.85
Site of relapse	1.47 (0.98–2.21)	0.07	0.85 (0.56–1.31)	0.47
HER2 status of metastasis	1.46 (0.50–4.28)	0.49	1.49 (0.34–6.52)	0.60

*HER2* human epidermal growth factor receptor 2

## Discussion

The development of resistance to endocrine therapy involves alteration of multiple pathways that may be targeted with novel therapeutic agents. Inhibition of growth factor receptor pathways that cross-talk with ER and blockage of cell cycle progression have been shown to be promising strategies in ER+ breast cancer treatment. A growing body of evidence implicating enrichment of EMT markers in breast cancer cells resistant to endocrine treatment supports the use of novel pharmacological strategies targeting EMT for breast cancer. By inhibiting EMT, tumor cells could maintain, or reverse to, an epithelial state with reduced migratory capacity and re-sensitization to endocrine therapy. However, the specific molecules involved in the regulation of EMT that should be targeted to overcome endocrine resistance remain to be defined.

In this study, we show that SNAI2, a mediator of EMT highly expressed in triple-negative breast cancer [26], is upregulated in endocrine-resistant cells, whereas other EMT-associated transcription factors, such as SNAI1/3, TWIST1/2, ZEB1/2, FOXC2, and GSC, are unaltered. We previously reported that miRNA-593, which is predicted to target SNAI2, is downregulated in tamoxifen-resistant cell lines, implicating SNAI2 in tamoxifen resistance [24]. We also showed that this EMT-inducing transcription factor is a key molecule in the control of tamoxifen-resistant cell growth [24]. In the present study, we focused on the possible role of SNAI2 in fulvestrant resistance and showed that fulvestrant-resistant cells, which express high levels of SNAI2, exhibit increased migration, higher expression of the mesenchymal marker vimentin, and reduced levels of the epithelial marker E-cadherin compared with the parental fulvestrant-sensitive cell line. Our data concur with previous studies showing that tamoxifen-resistant MCF-7 breast cancer cells display enhanced motile and invasive behavior and EM-like properties compared with tamoxifen-sensitive MCF-7 cells [27, 28].

Previous investigations have demonstrated that silencing of ER in ER+ MCF-7 breast cancer cells leads to acquisition of endocrine resistance and mesenchymal features, which contribute to tumor aggressiveness and metastatic ability [6, 29]. Studies have also reported that ER and SNAI2 levels are inversely correlated and that ER directly suppresses SNAI2 transcription, thus regulating EMT [30, 31]. Despite decreased ER levels in our fulvestrant-resistant cells expressing high SNAI2, these cells remain ER+ and are growth-stimulated by estrogen and inhibited by tamoxifen treatment [16]. Although overexpression of SNAIL in ER+ breast cancer cell lines has been shown to induce resistance to tamoxifen accompanied by reduced ER levels, ectopic expression of ER in these cells did not restore sensitivity to tamoxifen, suggesting that SNAIL might promote resistance to anti-estrogens independent of ER signaling [32].

We observed that reduction of SNAI2 expression impaired cell migration and increased E-cadherin levels in two fulvestrant-resistant breast cancer cell models, confirming a key role for SNAI2 in the control of cell motility and maintenance of a mesenchymal phenotype in resistant cells. These findings are in line with previous reports showing increased mesenchymal characteristics by ectopic expression of SNAI2 in ER+ MCF-7 cells [10]. Additionally, it has been shown that SNAI1/SNAI2 can induce drug resistance in breast cancer cells via alteration of cell survival signaling pathways [24, 32]. We demonstrated that siRNA-mediated knockdown of SNAI2 impairs growth of fulvestrant-resistant cells, which exhibit a high level of SNAI2. In contrast, growth of SNAI2-low breast cancer cells was significantly inhibited by fulvestrant alone, and downregulation of SNAI2 had no additional effect on decreasing the growth of these cells compared to standard endocrine therapy. The role of SNAI2 in controlling the growth of resistant cell lines was further supported by the observation that treatment with a chemical agent interfering with SNAIL binding to p53 (GN25) markedly decreased their growth. Although it is not clear that the growth inhibitory effect of GN25 was due to specific inhibition of SNAI2, as the agent targets other SNAI proteins such as SNAI1 and SNAI3, it seems plausible since SNAI2 was the only SNAI-family member that exhibited increased expression in fulvestrant-resistant cells. These findings suggest that tumor cells exhibiting high levels of SNAI2 may benefit from inhibition of SNAI2 in combination with standard fulvestrant treatment, while tumors with low SNAI2 expression can be treated with fulvestrant alone. Previous studies have demonstrated that SNAI2 increases stemness in breast cancer cells when co-expressed with SOX9 [33] or through regulation of stem cell markers, including c-Myc, SOX2 and Oct4 [10], possibly contributing to drug resistance. Interestingly, we found upregulation of SOX2 in MCF-7-based fulvestrant-resistant cells compared to fulvestrant-sensitive cells, and SNAI2 knockdown decreased SOX2 expression in three of the four fulvestrant-resistant cell lines, suggesting that SNAI2 might be involved in the mechanism of regulation of this stem marker in fulvestrant resistance.

Finally, we evaluated the clinical relevance of SNAI2 expression in metastatic lesions from two independent cohorts of patients with ER+ breast cancer treated with endocrine therapy in the advanced setting and showed that high SNAI2 levels correlated significantly with shorter PFS in patients on endocrine therapy, including fulvestrant. Correlation between high SNAI2 expression in primary breast tumors and shorter relapse-free survival has been previously demonstrated in ER+ breast cancer [34]. Studies have also shown that high SNAI2 expression in primary ER- breast tumors correlates with poor prognosis in those patients [35, 36]. Nevertheless, our study is the first to our knowledge to report the prognostic value of SNAI2 in patients with ER+ advanced breast cancer treated with endocrine therapy.

## Conclusions

In summary, our data support SNAI2 as a key regulator of the aggressive phenotype observed in endocrine-resistant breast cancer cells and a prognostic biomarker in ER+ advanced breast cancer treated with endocrine therapy. These findings highlight the role of SNAI2 as a potential target for therapeutic strategies against EMT and endocrine resistance.

## Additional files

Additional file 1: **Figure S1.** SNAI2 and E-cadherin protein levels following siRNA-mediated SNAI2 knockdown. MCF-7-based fulvestrant-resistant and parental-sensitive cells were transfected with siRNA against SNAI2 and SNAI2 (A) and E-cadherin (B) protein levels were evaluated 96 h following transfection, by Western blotting. β-actin was used as loading control. (TIF 357 kb)

Additional file 2: **Figure S2.** SOX2 knockdown reduces growth of fulvestrant-resistant breast cancer cells. (A) 182R-1 fulvestrant-resistant cells were transfected with siRNA against SOX2 leading to a reduction at the mRNA level, as evaluated by RT-qPCR. Gene expression was normalized using *PUM1*. (B) SOX2 knockdown resulted in decreased growth of fulvestrant-resistant cells as measured by crystal violet-based colorimetric assay. Cells were grown in medium containing fulvestrant. Experiment was performed in technical triplicates and results are shown with error bars representing mean ± standard deviation. (TIF 313 kb)

Additional file 3: **Figure S3.** Correlation between SNAI2 expression and PFS in patients with ER+ metastatic breast cancer from cohort 1 and 2 treated with fulvestrant. Kaplan-Meier plots evaluating PFS according to expression of SNAI2 in ER+ metastatic lesions from fulvestrant-treated patients from cohort 1 (A) and cohort 2 (B). A two-sided *p* value (*p < 0.05) was calculated using log-rank testing. (TIF 351 kb)

## Abbreviations

CI: Confidence interval
CSCs: Cancer stem cells
ECM: Extracellular matrix
EMT: Epithelial-mesenchymal transition
ER +: Estrogen receptor-positive breast cancer
FBS: Fetal bovine serum
FDR: False discovery rate
FFPE: Formalin-fixed paraffin-embedded
FulvR: Fulvestrant-resistant cells
HER2: Human Epidermal growth factor Receptor 2
HR: Hazard ratio
HRP: Horseradish peroxidase
MET: Mesenchymal-epithelial transition
OD: Optical density
OUH: Odense University Hospital
PFS: Progression-free survival
RT-qPCR: Reverse transcription (RT)-quantitative (q)PCR
siRNA: Small interfering RNA
TamR: Tamoxifen-resistant cells
