# Supplementary figures and images for: SNAI2 upregulation is associated with an aggressive phenotype in fulvestrant-resistant breast cancer cells and is an indicator of poor response to endocrine therapy in estrogen receptor-positive metastatic breast cancer
Source: Breast Cancer Res. 2018 Jun 19;20:60. doi: 10.1186/s13058-018-0988-9 (PMC6009053; doi:10.1186/s13058-018-0988-9)

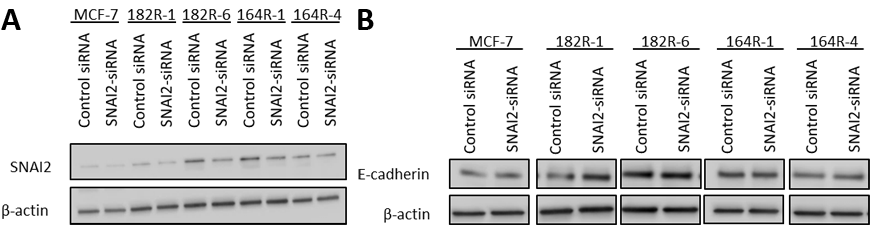

Supplement: Supplementary file 1 — Figure S1. SNAI2 and E-cadherin protein levels following siRNA-mediated SNAI2 knockdown. MCF-7-based fulvestrant-resistant and parental-sensitive cells were transfected with siRNA against SNAI2 and SNAI2 (A) and E-cadherin (B) protein levels were evaluated 96 h following transfection, by Western blotting. β-actin was used as loading control. (TIF 357 kb) [file 13058_2018_988_MOESM1_ESM.tif]

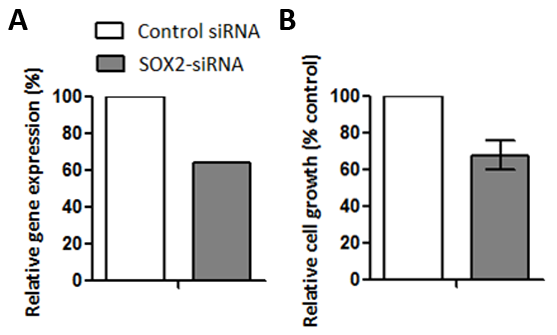

Supplement: Supplementary file 2 — Figure S2. SOX2 knockdown reduces growth of fulvestrant-resistant breast cancer cells. (A) 182R-1 fulvestrant-resistant cells were transfected with siRNA against SOX2 leading to a reduction at the mRNA level, as evaluated by RT-qPCR. Gene expression was normalized using PUM1. (B) SOX2 knockdown resulted in decreased growth of fulvestrant-resistant cells as measured by crystal violet-based colorimetric assay. Cells were grown in medium containing fulvestrant. Experiment was performed in technical triplicates and results are shown with error bars representing mean ± standard deviation. (TIF 313 kb) [file 13058_2018_988_MOESM2_ESM.tif]

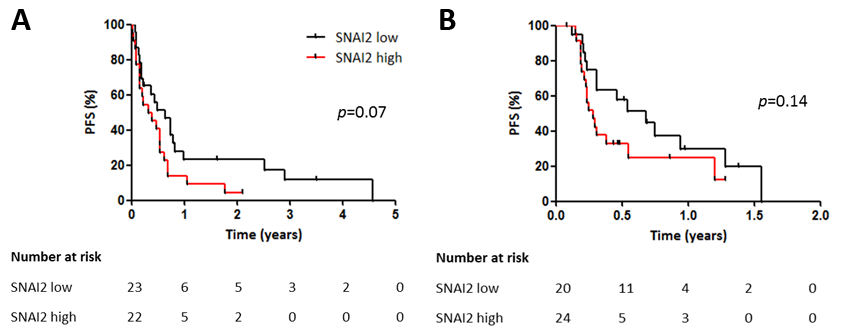

Supplement: Supplementary file 3 — Figure S3. Correlation between SNAI2 expression and PFS in patients with ER+ metastatic breast cancer from cohort 1 and 2 treated with fulvestrant. Kaplan-Meier plots evaluating PFS according to expression of SNAI2 in ER+ metastatic lesions from fulvestrant-treated patients from cohort 1 (A) and cohort 2 (B). A two-sided p value (*p < 0.05) was calculated using log-rank testing. (TIF 351 kb) [file 13058_2018_988_MOESM3_ESM.tif]
